# Li-Fraumeni syndrome with simultaneous osteosarcoma and liver cancer: Increased expression of a CD44 variant isoform after chemotherapy

**DOI:** 10.1186/1471-2407-12-444

**Published:** 2012-10-02

**Authors:** Go J Yoshida, Yasushi Fuchimoto, Tomoo Osumi, Hiroyuki Shimada, Seiichi Hosaka, Hideo Morioka, Makio Mukai, Yohei Masugi, Michiie Sakamoto, Tatsuo Kuroda

**Affiliations:** 1 Division of Gene Regulation, Institute for Advanced Medical Research, Keio University School of Medicine, 35 Shinanomachi, Shinjuku-ku, Tokyo, 1608582, Japan; 2 Division of Surgery, Department of Surgical Subspecialities, National Center for Child Health and Development, 2-10-1 Okura, Setagaya-ku, Tokyo, 1578535, Japan; 3 Department of Pediatrics, Keio University School of Medicine, 35 Shinanomachi, Shinjuku-ku, Tokyo, 1608582, Japan; 4 Department of Orthopedic Surgery, Keio University School of Medicine, 35 Shinanomachi, Shinjuku-ku, Japan; 5 Division of Diagnostic Pathology, Keio University Hospital, 35 Shinanomachi, Shinjuku-ku, Tokyo, 1608582, Japan; 6 Department of Pathology, Keio University School of Medicine, 35 Shinanomachi, Shinjuku-ku, Tokyo, 1608582, Japan; 7 Department of Pediatric Surgey, Keio University School of Medicine, 35 Shinanomachi, Shinjuku-ku, Tokyo, 1608582, Japan

**Keywords:** Li-Fraumeni syndrome (LFS), cancer stem cells (CSCs), CD44 variant isoforms

## Abstract

**Background:**

Li-Fraumeni syndrome (LFS) is a hereditary cancer predisposition syndrome that is commonly associated with a germline mutation in the tumor suppressor gene *p53*. Loss of p53 results in increased expression of CD44, a cancer stem cell (CSC) marker, which is involved in the scavenging of reactive oxygen species (ROS). Here, we report a change in the expression of a CD44 variant isoform (CD44v8-10) in an 8-year-old female LFS patient with osteosarcoma and atypical liver cancer after chemotherapy.

**Case presentation:**

The patient visited a clinic with a chief complaint of chronic pain in a bruise on her right knee. Magnetic resonance imaging (MRI) raised the possibility of a bone malignancy. Biochemical testing also revealed significantly elevated levels of AFP, which strongly suggested the existence of a primary malignancy in the liver. MRI imaging showed the simultaneous development of osteosarcoma and liver cancer, both of which were confirmed upon biopsy. Combined therapy with surgical resection after chemotherapy was successful in this patient. Regardless of the absence of a familial history of hereditary cancer, a germline mutation in *p53* was identified (a missense mutation defined as c.722 C>T, p.Ser241Phe). To better understand the cancer progression and response to treatment, immunohistochemical (IHC) analysis of biopsy specimens obtained before and after chemotherapy was performed using a specific antibody against CD44v8-10.

**Conclusion:**

This case demonstrates the ectopic up-regulation of CD44v8-10 in a biopsy sample obtained after cytotoxic chemotherapy, which confers high levels of oxidative stress on cancer cells. Because the alternative splicing of CD44 is tightly regulated epigenetically, it is possible that micro-environmental stress resulting from chemotherapy caused the ectopic induction of CD44v8-10 *in vivo*.

## Background

Li-Fraumeni syndrome (LFS) is a familial cancer predisposition syndrome, which is inherited in an autosomal dominant manner. This syndrome is most frequently associated with soft tissue sarcoma, osteosarcoma, pre-menopausal breast cancer, brain cancer, and adrenocortical carcinoma, but it can also result in other types of tumors. LFS is classified into two major subgroups: classic LFS and Li-Fraumeni-like (LFL) syndrome, which shares some, but not all, of the features of LFS [1-3]. Juvenile development of simultaneous and multiple cancers raises the possibility of LFS.

Sequence analysis of the entire *p53* coding region (exons 2–11) detects about 95% of *p53* mutations, most of which are missense mutations. A functional assay may be useful for determining the clinical significance of novel missense mutations [4]. It has been indicated that approximately 70% of LFS patients and 8–22% of patients with LFL syndrome have detectable *p53* mutations [5]. Comprehensive analyses of genotype-phenotype correlations have led to a better understanding of tumors that are associated with germline *p53* mutations [6].

CD44 is an adhesion molecule for extracellular matrix components such as hyaluronic acid and osteopontin [7], and plays an important role not only in wound healing and cell migration, but also in tumor invasion and metastasis. CD44 has numerous isoforms generated through alternative mRNA splicing. For instance, CD44v6 interacts with c-Met, the receptor of hepatocyte growth factor (HGF), thereby increasing the survival and proliferative ability of tumor cells That is one of the reasons why the expression of the CD44 splice variant CD44v6 is correlated with the metastasis of colon cancer to the liver and a poor clinical prognosis [8]. CD44 has been recently identified as one of the cellular surface markers associated with cancer stem cells (CSCs) in several types of tumors. Notably, CD44 variants (CD44v) are exclusively expressed in epithelial-type cells, whereas the CD44 standard isoform (CD44s) is expressed in both epithelial and mesenchymal cells [9].

Loss-of-functional mutations in the *p53* gene promote tumor development. CD44 expression is generally suppressed by *p53* binding to the *CD44* promoter, so that increased expression of CD44 is detected in tumor cells with mutant *p53*[10].

CSCs are defined as the small population of cancer cells with multi-lineage differentiation potential. These self-sustaining cells have the exclusive ability to self-renew and maintain the tumor tissue [11]. CSCs often fail to respond to chemotherapy, thereby causing distant metastasis and latent relapse. For this reason, we focused on the change in tumor expression of CD44 as a result of chemotherapy. Minimal residual disease (MRD) after chemotherapy is expected to be enriched in CSCs compared with the pre-chemotherapy tumor specimen.

Alternative splicing is mainly responsible for the diversity of CD44 isoforms. Among these variants, CD44v8-10 enhances reduced glutathione (GSH) synthesis by stabilizing the xCT transport system for cysteine, the precursor of GSH. Hence, CSCs acquire an enhanced reactive oxygen species (ROS) defense system [12]. Cytotoxic drugs such as Adriamycin induce apoptosis by causing oxidative stress, which damages DNA. To better understand the influence of oxidative stress on the tumor microenvironment, changes in the expression of CD44v8-10 were evaluated in response to chemotherapy. We hypothesized that oxidative stress due to the administration of cytotoxic chemotherapy would affect the expression of CD44v8-10 in *p53*-mutated cancer cells in this patient.

### Case presentation

**Figure 1:**
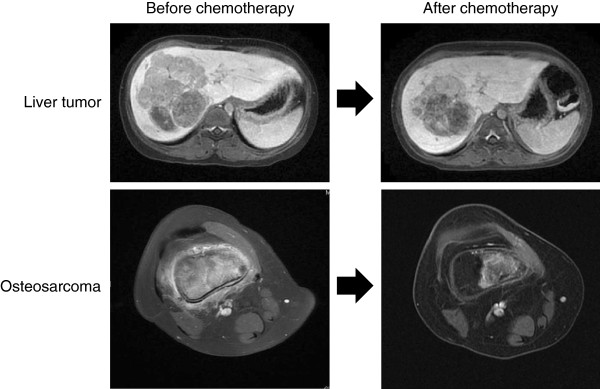
**Reduced tumor masses in the liver and bone after chemotherapy.** Magnetic resonance imaging (MRI) revealed that the sizes of both the liver tumor and the osteosarcoma were significantly reduced by the combination chemotherapy

**Figure 2:**
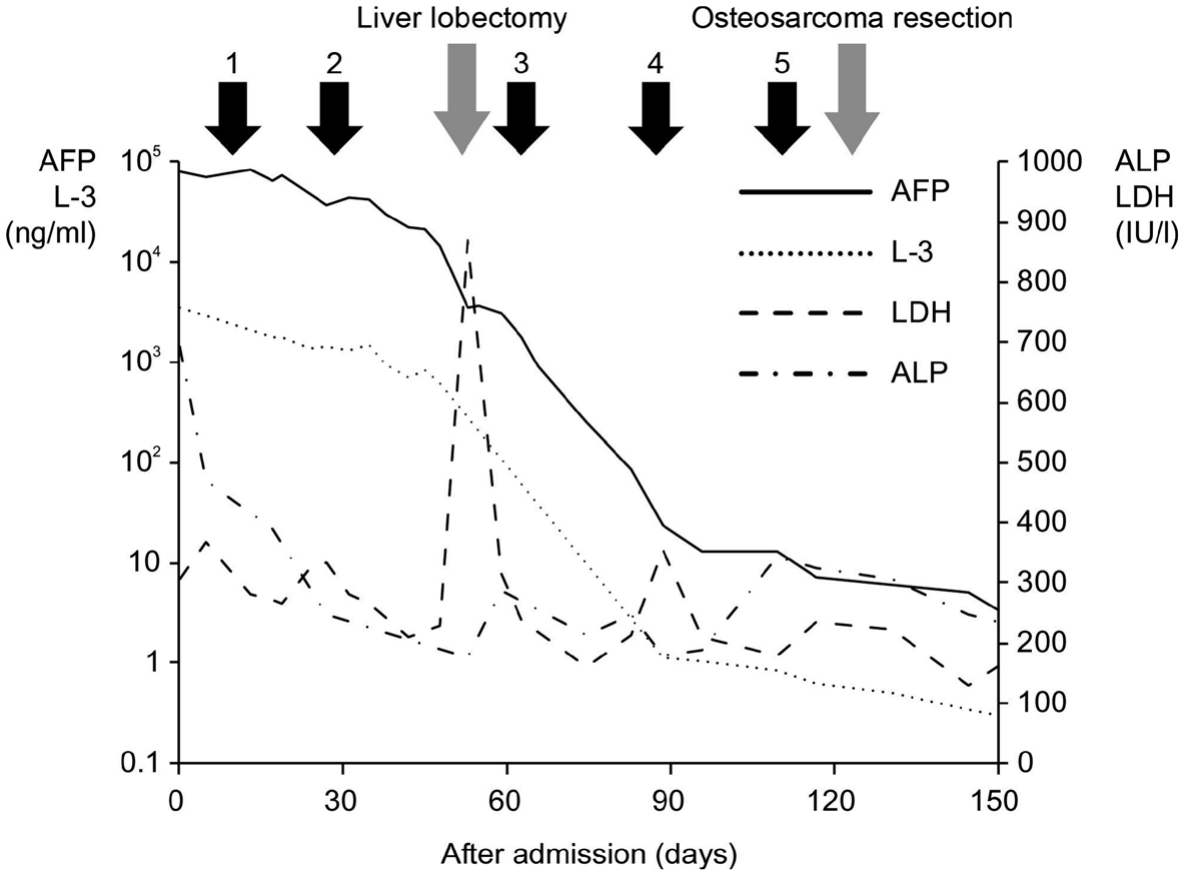
**Transitional serum biomarkers for liver cancer and osteosarcoma.** Combined therapy with surgical resection after chemotherapy resulted in a significant decrease in serum tumor markers for liver cancer and osteosarcoma. AFP and L-3 are serum markers for the hepatic tumor, whereas LDH and ALP are serum markers for the osteosarcoma

An 8-year-old female slipped on the stairs and had a bruise on her right knee. However, since the pain persisted, the patient visited a nearby orthopedic clinic. Magnetic resonance imaging (MRI) revealed the possibility of a bone malignancy (Figure 1). The patient was then admitted to the hospital for further examination. Biochemical testing revealed significantly elevated levels of both serum AFP (AFP: 79016 ng/ml, L3: 3555 ng/ml (4.5%), PIVKA-II: 128 IU/l), which suggested possible malignancy of the liver. A serum AFP level as high as 79,000 ng/ml was also indicative of a liver tumor. Antibodies against hepatitis virus B and C were not detected. The development of hepatoblastoma was atypical in terms of the age of onset, given that hepatoblastomas typically arise in patients under 3 years of age. Pathological diagnosis was transitional liver cell tumor (TLCT). A subset of liver cell tumors in older children and adolescents may develop as an intermediate between blastomatous tumors and adult-type tumors. This type of tumor, referred to as TLCT, is highly dependent on Wnt signaling than other categories of liver tumors [13]. MRI imaging showed the simultaneous development of osteosarcoma and liver cancer (Figure 1), both of which were confirmed by biopsy. After two courses of standard neoadjuvant chemotherapy for hepatoblastoma (a combination of cisplatin (CDDP) and tetrahydropyranyl-adriamycin (THP-ADR)), the residual liver tumor was removed completely by surgical extended lobectomy. After treatment with the standard neoadjuvant chemotherapy protocol for osteosarcoma (a combination of CDDP, ifosfamide (IFO), and methotrexate (MTX)), the diminished osteosarcoma was totally resected and an artificial joint was placed. The levels of transitional serum biomarkers for the liver tumor and osteosarcoma are shown (Figure 2), in which the blue arrows indicate repeated neoadjuvant chemotherapy. To date, there has been no sign of relapse or latent metastasis for about two years after last surgery. Combined therapy with surgical resection after chemotherapy was, therefore, successful in this patient. Given that we found the simultaneous development of a liver tumor and osteosarcoma, we performed genetic testing to evaluate the patient’s susceptibility to malignancy. Despite the absence of a familial history of hereditary cancer, a germline mutation in *p53* (c.722 C>T, p.Ser241Phe) was identified. Notably, the frequency of *de novo* mutations in LFS is between 7% and 20% [14]; it is, therefore, conceivable that the *p53* mutation occurred *de novo* during embryonic development.

**Figure 3:**
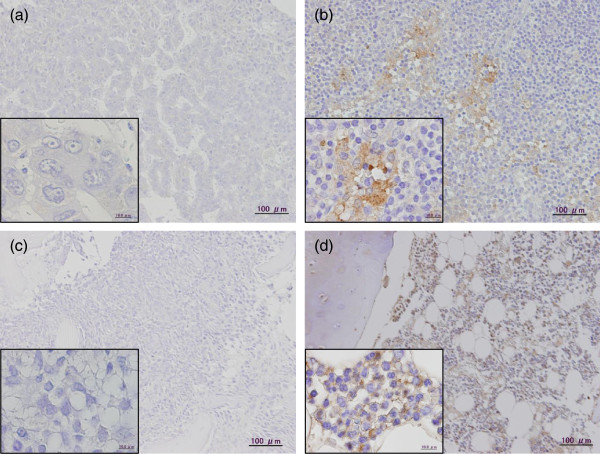
**CD44v8-10 IHC of both tumors before and after chemotherapy.** Immunohistochemical analysis of a transitional liver cell tumor (TLCT) prior to chemotherapy (**a**), a post-chemotherapy TLCT tissue (**b**), an osteosarcoma biopsy tissue sample obtained prior to chemotherapy (**c**), and a post-chemotherapy osteosarcoma tissue (**d**) stained with an antibody against CD44v8-10 (Inset: higher magnification; scale bar: 10 μm).(Inset: higher magnification; scale bar: 10 μm)

Immunohistochemical (IHC) analysis of the patient’s tumors prior to and following chemotherapy was performed using an antibody against the human CD44 variant isoform. CD44v8-10 was not expressed in both the liver tumor and the osteosarcoma before chemotherapy, but was ectopically expressed after chemotherapy (Figure 3).

### Discussion and conclusion

CD44 exists in as many as 16 different isoforms, which are generated through alternative mRNA splicing. CD44v8-10 is generated by epithelial splicing regulatory protein 1 (ESRP1), an RNA-binding protein [15]. Whereas the standard CD44 isoform (CD44s), which contains exons 1–5 and 16–20, is expressed predominantly in hematopoietic cells and normal epithelial cell subsets, CD44 variant isoforms with insertions in the membrane-proximal extracellular region are abundant in epithelial-type cancers, including liver tumors. Recently, CD44v8-10 was reported to have a novel function; it inhibits oxidative stress in tumor cells by promoting the GSH synthesis [12]. However, exactly transcriptional factors or epigenetic mechanisms controlling the induction of ESRP1, the master regulator of CD44v8-10, remain unclear [9]. We have already performed IHC using the specific antibody against human ESRP1 and ESRP2. Unfortunately, there was no antibody which was suitable for IHC due to reactions to the non-specific antigens, so that we could not obtain data that shows ESRP1 or ESRP2 exclusively in the nucleus (data not shown).

Osteosarcoma originates from mesenchymal tissue, not epithelial tissue, meaning that the CD44 variant isoforms expressed in osteosarcoma may have a completely different function from those expressed in liver cancer. Whereas the function of CD44v8-10 in osteosarcoma is unknown, the only v6 expression is negatively correlated with 5-year metastasis-free survival. Therefore, overexpression of the variant isoform, CD44v6, is considered a poor prognostic marker in patients with osteosarcoma [15]. However, no specific data has been reported regarding the biological significance of CD44v8-10 in osteosarcoma. The cytotoxic drugs CDDP and THP-ADR increase the ROS level in cancer cells, thereby inducing DNA damage and apoptosis. It is possible that CD44v8-10 is induced following chemotherapy in osteosarcoma to counteract this oxidative stress.

Considering the lack of familial history of malignancy in this case, it is supposed that the de novo p53 germline mutation occurred during early development. The p53 protein suppresses expression of CD44 by binding to the promoter region of CD44 [10], and CD44v8-10 was not expressed in both tumors before chemotherapy. The absence of p53 likely enabled the upregulation of CD44v8-10 in response to chemotherapy. However, this finding does not rule out the possibility that other CD44 isoforms were fully expressed prior to chemotherapy. Although cancer cells may acquire resistance to oxidative damage by upregulating CD44v8-10, there has been no sign of relapse or metastasis so far in this patient. The fact that CD44v8-10 expression was not correlated with increased malignant potential in this case seems to be contradictory to our previous research [12]; however, the effect of CD44v8-10 expression may depend on the origin of the tumor. It is possible that CD44v8-10 may function differently in hepatic tumors and osteosarcomas with p53 mutations than in gastric and colorectal cancers.

**Figure 4:**
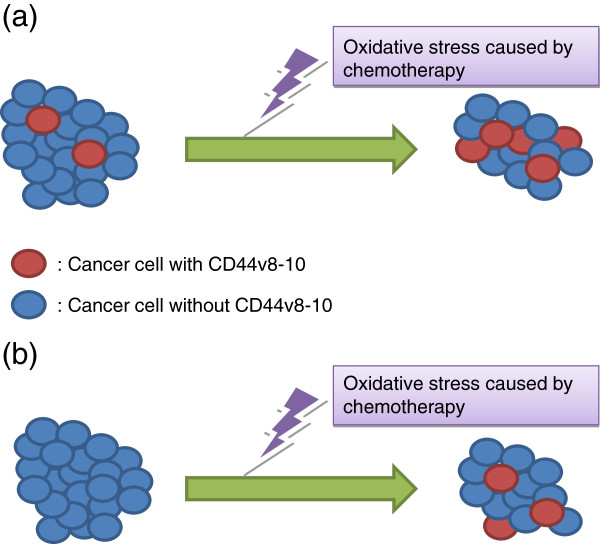
**Two hypothetical models for the induction of CD44v8-10 after chemotherapy.** Cancer cells expressing CD44v8-10 are present in the original tumor and clonally expand in response to anti-cancer drugs (**a**). Cancer cells with CD44v8-10 are absent from the original tumor, but oxidative stress caused by chemotherapy induces the ectopic expression of CD44v8-10 (**b**)

Potential mechanisms for the ectopic induction of CD44v8-10 expression in osteosarcoma are shown (Figure 4):

a) Cancer cells expressing CD44v8-10 may have existed in the original tumor, but were too rare to be detected by IHC. These cells survived even under conditions of oxidative stress caused by cytotoxic chemotherapy.

b) Cancer cells expressing CD44v8-10 did not exist originally, but the oxidative stress caused by chemotherapy induced the expression of CD44v8-10 in the tumor.

Differentiating between these possibilities will require further investigation into how the alternative splicing machinery for CD44 is affected by chemotherapy. There has been no sign of relapse or metastasis so far in this patient, but it will be crucial to gather additional data regarding CSC molecular dynamics during the course of chemotherapy. Therefore, a larger number of cancer cases need to be analyzed, and longer-term follow-up studies must be conducted, to confirm the results of this study.

### Consent

Written informed consent was obtained from the patient for publication of this case report and the accompanying images. A copy of the written consent is available for review upon request.

## Abbreviations

(LFS): Li-Fraumeni syndrome; (CSC): Cancer stem cell; (ROS): Reactive oxygen species; (MRI): Magnetic resonance imaging; (LFL): Li-Fraumeni-like; (GSH): Glutathione; (MRD): Minimal residual disease; (TLCT): Transitional liver cell tumor; (IHC): Immunohistochemical; (ESRP1): Epithelial splicing regulatory protein 1; (CDDP): Cisplatin; (THP-ADR): Tetrahydropyranyl-adriamycin.

## Competing interests

The authors declare that they have no competing interests to disclose.

## Authors’ contribution

GJY: carried out the immunological staining analysis, and reviewed the literature, drafted and edited the manuscript, YF, TO, HS, SH and HM: were involved in the patient active management , drafted and edited the manuscript, MM, YM and MS: processed and provided pathology, TK: critically revised the manuscript. All authors read and approved the final manuscript.

## Pre-publication history

The pre-publication history for this paper can be accessed here:

http://www.biomedcentral.com/1471-2407/12/444/prepub
